# Quantitative PSMA PET Biomarkers for Predicting Response to ^177^Lu-PSMA Therapy in Prostate Cancer: A Systematic Review and Meta-analysis

**DOI:** 10.2967/jnumed.125.270872

**Published:** 2026-06

**Authors:** Iman Kiani, Mohammad Amin Siri, Mahan Babaei, Mohammad Mahdi Aliasghari, Saeed Mohammadzadeh, Sara Mohammadi, Ali Salavati

**Affiliations:** 1 Students’ Scientific Research Center, Tehran University of Medical Sciences, Tehran, Iran;; 2 School of Medicine, Shahid Beheshti University of Medical Sciences, Tehran, Iran;; 3 School of Medicine, Tehran University of Medical Sciences, Tehran, Iran; and; 4 Department of Nuclear Medicine and Theranostics, David Geffen School of Medicine, UCLA, Los Angeles, California

**Keywords:** PSMA PET, mCRPC, prostate cancer, ^177^Lu-PSMA therapy

## Abstract

^177^Lu-labeled prostate-specific membrane antigen (PSMA) radiopharmaceutical therapy (^177^Lu-PSMA-617) has emerged as a promising targeted therapy for metastatic castration-resistant prostate cancer (mCRPC). However, response rates to therapy vary among patients. Quantitative PSMA PET biomarkers have been increasingly explored for their prognostic potential in guiding patient selection. This systematic review and meta-analysis aims to evaluate the prognostic value of PSMA PET biomarkers for response to ^177^Lu-PSMA therapy in patients with mCRPC. **Methods:** A systematic search was conducted across PubMed, Scopus, Web of Science, and Embase databases to identify studies evaluating quantitative PSMA PET parameters as predictors of response to ^177^Lu-PSMA therapy. Hazard ratios (HRs) and 95% CIs for quantitative PSMA PET parameters were extracted and synthesized using random-effects meta-analysis. Heterogeneity was assessed using *I*^2^ statistics, and publication bias was evaluated via funnel plots and the Egger test. **Results:** A total of 23 studies met the inclusion criteria. Among PSMA PET parameters, SUV_mean_, tumor volume (TV), or total lesion (TL) PSMA emerged as significant predictors of overall survival (OS), progression free survival, and prostate-specific antigen response. The pooled HR for SUV_mean_ in predicting OS was 0.93 (95% CI, 0.90–0.96; *I*^2^ = 92.8%), whereas TV showed a HR of 1.37 (95% CI, 1.27–1.47; *I*^2^ = 4.7%), and the TL had a HR of 1.04 (95% CI, 1.03–1.05; *I*^2^ = 16.1%). Additionally, SUV_max_ and SUV_peak_ demonstrated limited predictive value. **Conclusion:** Our findings indicate that SUV_mean_, TV, and TL derived from PSMA PET imaging could serve as valuable biomarkers for prognosticating response to ^177^Lu-PSMA therapy in patients with mCRPC. Future studies should aim to integrate PET biomarkers with clinical and biochemical parameters to improve treatment decision-making.

## Visual Abstract

**Figure:**
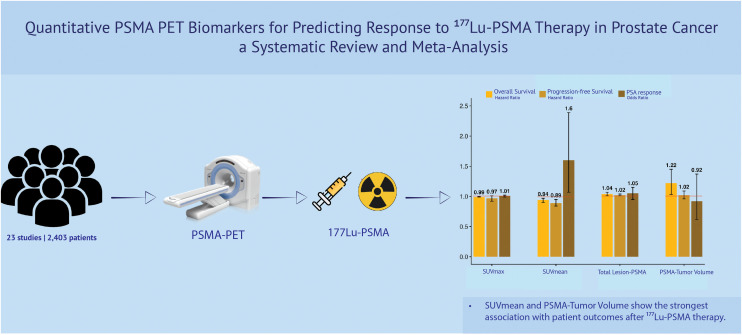

Prostate cancer is one of the most prevalent malignancies worldwide (1). According to the World Health Organization, it was the fourth most commonly diagnosed cancer in 2022, accounting for 1,467,854 cases (7.3% of all cancer cases) and ranking as the eighth leading cause of cancer-related deaths globally (2). At an advanced stage, metastatic castration-resistant prostate cancer (mCRPC) could develop, characterized by resistance to androgen deprivation therapy (3). Although multiple systemic treatments are now available, including androgen receptor signaling inhibitors, taxane-based chemotherapy, and targeted radiopharmaceutical therapy (RPT), clinical outcomes remain heterogeneous (4). Therefore, identifying prognostic biomarkers is important for individualizing therapy, improving survival outcomes, and minimizing unnecessary toxicity in patients with advanced disease.

Traditionally, the treatment of mCRPC has been primarily limited, with a slight impact on overall survival (OS) (5). However, recent advances in prostate-specific membrane antigen (PSMA) targeted RPT have transformed the treatment landscape. One of the most promising therapies is ^177^Lu (^177^Lu-PSMA-617), a targeted RPT that selectively binds to PSMA-expressing cancer cells. This approach enables β-emitting radiation to effectively reduce tumor burden while minimizing damage to healthy tissues, ultimately leading to improved OS in patients with mCRPC (6). ^177^Lu-PSMA therapy has been approved for patients with PSMA-positive mCRPC (7,8).

Given the high costs associated with ^177^Lu-PSMA RPT and potential treatment-related toxicities, careful patient selection becomes important to optimize clinical outcomes and justify health care resource allocation. Indeed, economic analyses have shown that ^177^Lu-PSMA RPT in appropriately selected patients provides notable long-term clinical benefits, as measured in quality-adjusted life years, and is cost-effective in specific clinical scenarios (9). Moreover, although ^177^Lu-PSMA RPT has demonstrated meaningful survival benefits in appropriately selected patients, it may also be associated with adverse effects such as xerostomia, hematologic toxicity, and renal impairment, which can significantly impact quality of life in this palliative setting (10). Therefore, this systematic review aims to identify patient subgroups that derive the greatest benefit from ^177^Lu-PSMA therapy based on pretreatment PSMA PET biomarkers and to determine the clinical contexts in which this treatment offers the most significant advantages.

## MATERIALS AND METHODS

This systematic review and meta-analysis adhered to the Preferred Reporting Items for Systematic Reviews and Meta-Analyses (PRISMA) guidelines. The protocol for this review was preregistered in PROSPERO (ID: CRD42025637610).

### Search Strategy and Study Selection

A comprehensive literature search was conducted using PubMed, Scopus, Web of Science, and Embase to identify relevant studies published up to January 15, 2025. The search incorporated a combination of Medical Subject Headings terms and free-text keywords related to PSMA PET, ^177^Lu-PSMA therapy, prostate cancer, RPT, and treatment response prognostication. To design the search strategy, 2 reviewers independently devised a strategy and then merged the strategies. If there was a discrepancy, the third reviewer was consulted. The finalized search strategy is provided in Supplemental Table 1 (supplemental materials are available at http://jnm.snmjournals.org). To ensure thorough coverage, the reference lists of included articles and relevant systematic reviews were also manually screened for additional eligible studies.

Studies were considered eligible for inclusion if they investigated PSMA PET biomarkers as predictors of response to ^177^Lu-PSMA therapy and reported at least 1 quantitative PET parameter such as SUV_max_, SUV_mean_, SUV_peak_, tumor volume (TV), or total lesion (TL) PSMA. Additionally, studies had to provide hazard ratios (HRs), odds ratios (ORs), or correlation coefficients with 95% CIs for oncologic and biochemical endpoints and report at least 1 of the following endpoints: OS, progression-free survival (PFS), or prostate-specific antigen (PSA) response.

Studies were excluded if they were case reports, reviews, conference abstracts, editorials, or expert opinions. Furthermore, studies that did not include quantitative PET parameter analysis or relevant clinical outcome data were also excluded. Non-English publications were omitted unless a full translation was available.

The selection process was conducted by 2 independent reviewers who screened the titles and abstracts of identified records. Full-text articles were then assessed for eligibility, and any disagreements regarding study inclusion were resolved through discussion with a third reviewer.

### Data Extraction and Quality Assessment

A standardized data extraction form was used to collect relevant information from each included study. Extracted data included study characteristics (first author, publication year, study design, sample size, and country), patient characteristics (age, disease stage, prior treatments, and PSMA PET imaging protocols), quantitative PET parameters (SUV_max_, SUV_mean_, SUV_peak_, TV, TL), and clinical outcomes (OS, PFS, PSA response). Additionally, statistical metrics such as HRs, ORs, CIs, and *P* values for PET biomarkers were recorded.

The quality of included studies was assessed using the Prediction Model Risk of Bias Assessment Tool, which evaluates risk of bias across 4 domains: participants, predictors, outcomes, and analysis methods. Each study was classified as having low, moderate, or high risk of bias based on predefined criteria.

### Statistical Analysis

A random-effects meta-analysis was conducted to pool HRs using the DerSimonian and Laird method (11) to pool HRs and evaluate the prognostic value of PSMA PET biomarkers for prognosticating response to ^177^Lu-PSMA therapy. Heterogeneity among studies was assessed using *I*^2^ statistics, with values of 0%–25% indicating low heterogeneity, 26%–50% indicating moderate heterogeneity, and greater than 50% indicating high heterogeneity.

Subgroup analyses were conducted to explore variations based on PET-derived parameters (SUV_max_, SUV_mean_, SUV_peak_, TV, TL), various outcomes and PET imaging regions wherever data were adequate. To assess potential publication bias, funnel plots and the Egger test were used when more than 10 studies were present, with a *P* value of less than 0.05 considered indicative of significant bias. All statistical analyses were performed using STATA 17.0.

## RESULTS

### Search Results and Study Characteristics

**FIGURE 1.**
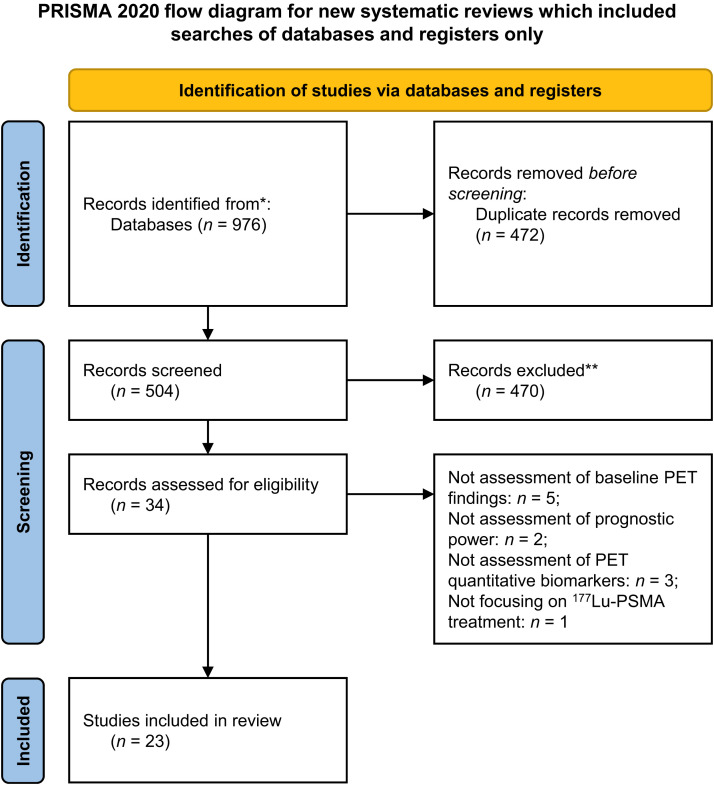
PRISMA flowchart. *Records were identified through systematic searches of electronic databases, including PubMed, Embase, Web of Science, and Scopus. **Records were excluded at title and abstract screening stage.

The initial database search yielded 976 records. After removing 472 duplicates, 504 records remained for screening based on titles and abstracts. Of these, 470 were excluded for not meeting the inclusion criteria. Full texts of the remaining 34 studies were assessed for eligibility. Eleven studies were excluded at this stage due to the following reasons: 5 did not assess baseline PET findings, 2 lacked assessment of prognostic power, 3 did not analyze quantitative PET biomarkers, and 1 did not focus on ^177^Lu-PSMA therapy (Supplemental Table 2). Ultimately, 23 studies met all inclusion criteria and were included in the final review (Fig. 1) (12–34).

The included studies had a range of study designs, settings, and patient cohorts. Sample sizes varied from 20 to 414 patients with a total of 2,403 patients. The majority of studies were conducted in single-center, retrospective settings. The mean age of participants across studies typically ranged between late 60s and early 70s. A variety of PSMA PET–derived imaging biomarkers were assessed, including SUV_max_, SUV_mean_, TV PSMA, PSMA TL, and derived ratios such as tumor-to-liver uptake. Endpoints most commonly reported included PSA response, PFS, and OS. Prior medication usage was heterogeneous but commonly involved androgen deprivation therapy, androgen receptor signaling inhibitors (abiraterone, enzalutamide), taxane chemotherapy (docetaxel, cabazitaxel), and across multiple studies, SUV_mean_ and SUV_max_ were frequently reported as prognostic of treatment response (Supplemental Table 3).

### Risk of Bias Assessment

Table 1 provides a comprehensive assessment of the risk of bias based on Prediction Model Risk of Bias Assessment Tool criteria. Most studies were rated as having a low risk of bias across the key domains of participants, predictors, and outcomes. However, in the analysis domain, a few studies did not avoid relying solely on univariate analysis, which may limit the robustness of their prognostic findings.

**TABLE 1.** Quality Assessment of Included Studies

	ROB				Applicability			Overall	
Study	Participants	Predictors	Outcome	Analysis	Participants	Predictors	Outcome	ROB	Applicability
Aksu et al. (2022)	Low	Low	Low	Unclear	Low	Low	Low	Low	Low
Assadi et al. (2022)	Low	Low	Low	Low	Low	Low	Low	Low	Low
Crumbaker et al. (2023)	Low	Low	Low	Unclear	Low	Low	Low	Low	Low
Emmett et al. (2019)	Unclear	Low	Low	Unclear	Unclear	Low	Low	Low	Low
Emmett et al. (2023)	Low	Low	Low	Unclear	Low	Low	Low	Low	Low
Erdogan et al. (2022)	Low	Low	Low	Unclear	Low	Low	Low	Low	Low
Ferdinandus et al. (2017)	Low	Low	Low	Low	Low	Low	Low	Low	Low
Ferdinandus et al. (2020)	Low	Low	Low	Unclear	Low	Low	Low	Low	Low
Gafita et al. (2021)	Low	Low	Low	Low	Low	Low	Low	Low	Low
Gafita et al. (2024)	Low	Low	Low	Low	Low	Low	Low	Low	Low
Groener et al. (2023)	Low	Low	Low	Low	Low	Low	Low	Low	Low
Hartrampf et al. (2023)	Low	Low	Low	Low	Low	Low	Low	Low	Low
Hein et al. (2024)	Low	Low	Low	Low	Low	Low	Low	Low	Low
Hotta et al. (2023)	Low	Low	Low	Low	Low	Low	Low	Low	Low
Huang et al. (2021)	Low	Low	Low	Low	Low	Low	Low	Low	Low
Kuo et al. (2024)	Low	Low	Low	Low	Low	Low	Low	Low	Low
Rosar et al. (2024)	Low	Low	Low	Low	Low	Low	Low	Low	Low
Seifert et al. (2021)	Low	Low	Low	Low	Low	Low	Low	Low	Low
Seifert et al. (2021)	Low	Low	Low	Unclear	Low	Low	Low	Low	Low
Swiha et al. (2024)	Low	Low	Low	Low	Low	Low	Low	Low	Low
Wang et al. (2023)	Low	Low	Low	Low	Low	Low	Low	Low	Low
Widjaja et al. (2023)	Low	Low	Low	Unclear	Low	Low	Low	Low	Low
van der Sar et al. (2022)	Low	Low	Low	Unclear	Low	Low	Low	Low	Low

ROB = risk of bias.

### OS Analysis

**FIGURE 2.**
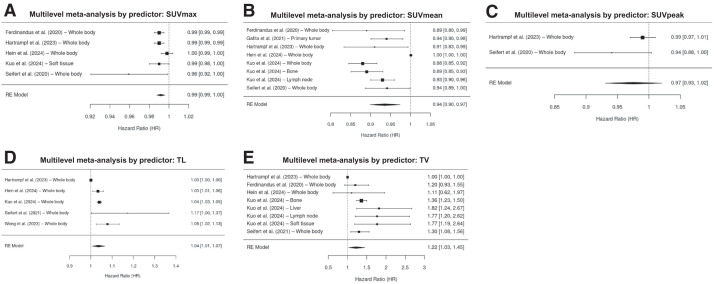
Forest plot of HR values for all PET/CT predictors of OS.

In total, 7 patient studies were included in the analysis (19,20,23,24,27,29,30). Overall, 20 individual PSMA PET predictors of OS in patients with mCRPC were identified. SUV_mean_, TV, TL, and SUV_max_ were the most frequently reported OS predictors, with TV being the most dominant prognostic factor in determining OS. The meta-analysis revealed a HR of 0.93 (95% CI, 0.90–0.96; *I*^2^ = 93.4%) for SUV_mean_, 1.37 (95% CI, 1.27–1.48; *I*^2^ = 17.1%) for TV, 1.04 (95% CI, 1.03–1.05; *I*^2^ = 32.8%) for TL, and 0.99 (95% CI, 0.99–1.00; *I*^2^ = 49.3%) for SUV_max_. The forest plot in Figure 2 shows pooled HR values for all PSMA PET predictors of OS.

Region-based analysis is depicted in the supplemental materials. Whole-body PET/CT, the most prevalent examination, revealed a HR of 0.99 (95% CI, 0.96–1.02). In contrast, PET/CT evaluations on soft tissue, lymph nodes, liver, and bone demonstrated more promising outcomes with HR values of 1.28 (95% CI, 0.73–2.25), 1.24 (95% CI, 0.66–2.33), 1.82 (95% CI, 1.24–2.67), and 1.10 (95% CI, 0.72–1.66) (Supplemental Fig. 1).

### PFS Analysis

**FIGURE 3.**
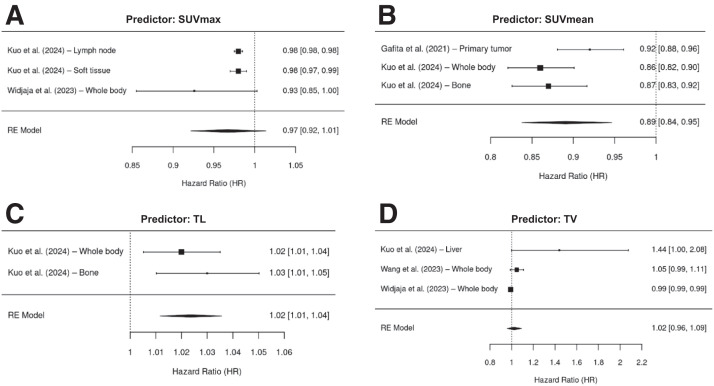
Forest plot of HR values for all PET/CT predictors of PFS.

Four studies were included in the analysis (20,27,33,34). In total, 12 distinct PET/CT predictors of PFS in patients with mCRPC were identified. The pooled HR value was 0.88 (95% CI, 0.85–0.92) for SUV_mean_, 1.02 (95% CI, 0.96–1.09) for TV, 1.02 (95% CI, 1.01–1.04) for TL, and 0.98 (95% CI, 0.97–0.99) for SUV_max_. Pooled HR values for the PET/CT predictor of PFS are displayed in the forest plot in Figure 3. When evaluating each PET/CT examination in each region, whole-body PET/CT, as the most common examination, indicated a pooled HR of 0.97 (95% CI, 0.90–1.04), and PET/CT evaluation on the liver demonstrated a HR of 1.44 (95% CI, 1.00–2.08). Detailed subgroup analyses based on anatomic sites of metastasis (e.g., lymph node, bone, visceral) are available in Supplemental Figure 2.

### PSA Response Assessment Analysis

**FIGURE 4.**
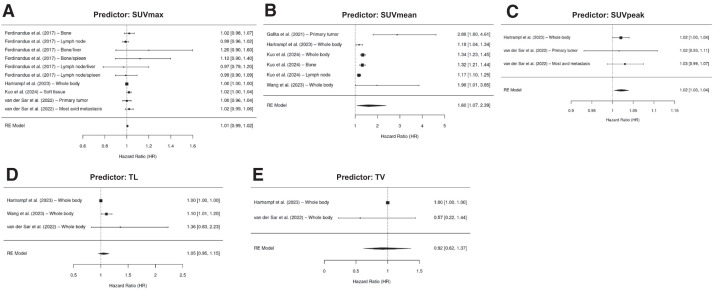
Forest plot of HR values for all PET/CT predictors of PSA response status.

Six papers with patients were included in the analysis (18,20,23,27,32,34). In total, 21 distinct PET/CT predictors were found to predict PSA response status in patients with mCRPC. The pooled OR value was 1.40 (95% CI, 1.14–1.71) for SUV_mean_, 1.02 (95% CI, 1.00–1.04) for SUV_peak_, and 1.01 (95% CI, 0.99–1.02) for SUV_max_. Pooled OR values for PET/CT predictor of PSA response status are represented in the forest plot of Figure 4. Analyses of PSMA PET biomarkers stratified by anatomic regions of metastasis are provided in Supplemental Figure 3.

### Score Development

Three studies proposed composite scores to enhance outcome prognostication. Rosar et al. (28) developed a Dual Imaging Progression Prediction score incorporating PSMA SUV_max_ and the ^18^F-FDG SUV_max_/PSMA-11 SUV_max_ quotient. At the optimal threshold and in combination with clinical findings, the score achieved a sensitivity of 88.2% and specificity of 87.5% for prognosticating lesion progression. Moreover, Swiha et al. (31) developed a semiquanitative score based on heterogeneity and SUV_max_ of the tumor. Their score, derived from tools available on a standard PET workstation, demonstrated prognostic value for both PFS and OS, with performance comparable to that of SUV_mean_. Lastly, Hotta et al. (25) developed a score based on quantitative and visual PSMA PET tumor–to–salivary gland ratio, which is unlike most prior studies that used liver uptake as the reference background. They found that PSA response rates were significantly greater in the high PSG score groups (69.6% for quantitative PSG and 63.2% for visual PSG) compared with intermediate (38.7% and 33.3%) and low score groups (16.7% and 16.1%) (*P* < 0.001 for both), with similar trends observed for PSA PFS and OS (25).

## DISCUSSION

This systematic review and meta-analysis demonstrates that quantitative PSMA PET/CT biomarkers are associated with treatment outcomes after ^177^Lu-PSMA therapy in patients with mCRPC. Across included studies, high PSMA TV was the frequently reported predictor of poor OS (HR, 1.37; 95% CI, 1.27–1.47), whereas SUV_mean_ was significantly associated with both OS and PFS.

RPT with ^177^Lu-PSMA is a relatively novel and promising option for addressing mCRPC, providing opportunities for specific tumor elimination (35). However, a significant number of patients may not benefit from this therapy. As reported by the VISION trial (36), although the proportion of patients with at least 50% PSA reduction was higher in the treatment group, more than half of the treatment group did not have this reduction. This indicates the need for optimized patient enrollment strategies for RPT. Since PSMA PET/CT is widely being used in the setting of mCRPC, assessing its ability to predict if patients will benefit from ^177^Lu-PSMA therapy can significantly aid physicians in providing the most appropriate therapeutic option. This, in turn, will hopefully result in improving mCRPC prognosis and establishing new guidelines regarding patient selection.

Generally, the included studies reported OS, PFS, and PSA reduction as the primary outcomes indicating the response to RPT. However, it should be declared that the dependency of the OS and PFS on the duration of the follow-up period can lead to heterogeneity. Moreover, PFS can be evaluated based on radiologic or biochemical findings. In this regard, Kuo et al. (27) reported radiographic PFS, whereas other authors (20,33,34) assessed PFS based on the PSA response. Some other factors could be responsible for heterogeneity among the included studies, namely, radioligand dose and cycles, isotopes implemented for imaging, site and number of metastases, current and previous treatments, time since the initial diagnosis of the tumor, and clinical and laboratory status of the patients. Taken together, future studies should focus on pursuing a unified strategy regarding outcome assessment and patient enrollment to improve the reliability of the drawn conclusions.

The most common quantitative factors evaluated using a PSMA PET/CT scan for mCRPC in the included studies were SUV_max_, SUV_mean_, SUV_peak_, and volumetric PET measurements (including TV and TL). SUV_max_ has been used for strict patient enrollment for ^177^Lu-PSMA-617 therapy in the TheraP trial (37). A recent meta-analysis conducted by Chen et al. (38) revealed that the prostatic lesion’s SUV_max_ in preoperative PSMA PET was correlated with the pathologic grade of the tumor. Moreover, higher SUV_max_ was associated with earlier biochemical recurrence. Nevertheless, high inconsistency was evident between the included studies, leading to authors concluding that SUV_max_ alone should not be used for clinical settings. This is in concordance with our study, in which SUV_max_ and SUV_peak_ were not significant predictors of OS, suggesting that maximum uptake values alone may not reliably reflect tumor burden or treatment response.

Also, regarding PFS, SUV_max_ had a near-neutral effect (HR, 0.98; 95% CI, 0.97–0.99), indicating limited prognostic utility. On the other hand, SUV_mean_ tended to be a predictor biomarker for OS (HR, 0.93; 95% CI, 0.90–0.96), PFS (HR, 0.88; 95% CI, 0.85–0.92), and PSA response (HR, 1.34; 95% CI, 1.18–1.54). This may indicate that patients with mCRPC with a higher SUV_mean_ are better candidates for ^177^Lu-PSMA therapy since there is a higher chance for radioligand binding. The superiority of SUV_mean_ may be justified, as unlike SUV_max_, this parameter considers every voxel of a specified region. However, determining the outline of an area is operator-dependent, which may cause variability in calculating SUV_mean_ (39). Recent advances in artificial intelligence–assisted 3-dimensional segmentation (deep learning and hybrid rule–based pipelines) can reduce this limitation by lowering operator dependence and standardizing lesion contouring (40). The integration of such automated, quality-controlled segmentation into quantitative PET workflows could enhance intercenter consistency and facilitate wider clinical adoption of PSMA-based imaging biomarkers.

On the prognostic performance of SUV_mean_, it is worth mentioning that a higher whole-tumor SUV_mean_ on PSMA PET likely reflects not only greater overall ligand availability but also more uniform PSMA expression throughout the lesion volume (31). From a radiobiologic standpoint, such homogeneous target distribution enables more even ^177^Lu-PSMA delivery, a higher absorbed dose per voxel, and fewer undertreated cold regions that could allow tumor regrowth. In contrast, lesions with low mean uptake but high SUV_max_ suggest patchy tracer distribution, dose heterogeneity, and a higher likelihood of radioresistant subclones. Importantly, SUV_max_ alone represents the single hottest voxel and thus fails to capture the broader biologic behavior of disease across the whole tumor burden, overlooking both intra- and interlesional heterogeneity that critically influence therapy response and progression patterns (41).

Volumetric parameters of PET have been studied in other cancers. For instance, meta-analyses revealed their prognostic role in esophageal, non–small cell lung, and cervical cancers (42–44). However, knowledge regarding PET volumetric parameters in the cases of mCRPC has yet to develop. In this regard, lower TV was associated with more favorable OS in 2 of 10 studies included in a meta-analysis (45). Similarly, in our analysis, higher TV was associated with more unfavorable OS (HR, 1.37; 95% CI, 1.27–1.47). TL is another PSMA PET/CT parameter, calculated by the following formula: ∑volume × SUV_mean_. This biomarker evaluates the total tumor burden by considering every metastasis in the whole body (46). Burgard et al. and Rosar et al. introduced this factor as an OS predictor in patients with mCRPC undergoing ^177^Lu-PSMA therapy (46,47). Our study revealed that TL may be a predictor for the 3 outcomes mentioned; however, the available studies assessing this parameter are extremely limited, warranting further investigations in this regard.

Noteworthy, in OS prognostication, we observed that TL had a better performance compared with SUV_mean_. As an explanation, biologically, larger disseminated mass implies greater clonogenic cell load (higher probability of resistant subpopulations), more micrometastatic niches with sublethal dosing, and systemic effects of disease burden (cachexia, organ compromise) (48). Because TL couples intensity to volume, it better maps to total activity required and achievable dose coverage, explaining its tighter association with OS than intensity alone. Nevertheless, future studies should elaborate on this aspect.

It should be declared that PSMA is overexpressed in malignant prostatic tissue, correlating with the aggressive behavior of the tumor (49). Therefore, PSMA has been targeted for achieving tumor elimination during RPT. Nevertheless, PSMA expression is not confined to the malignant prostatic tissue (50). Physiologic uptake in certain visceral organs, such as the spleen, liver, and salivary glands, can obscure lesion visibility and complicate interpretation (51). Moreover, variable or low PSMA expression within specific metastatic sites, compounded by partial-volume effects in small, necrotic, or dedifferentiated lesions, may lead to underestimation of disease burden (52,53). These so-called “PSMA-negative” or low-visibility metastases may still harbor viable tumor cells with high proliferative and metastatic potential, ultimately contributing to treatment resistance or early relapse despite apparently favorable imaging profiles (51). On the other hand, ^18^F-FDG PET highlights metabolic activity driven by glycolysis, which tends to be upregulated in more aggressive or dedifferentiated tumor clones (54), and it has been observed that ^18^F-FDG PET could be superior over PSMA PET in predicting adverse clinical outcomes in such contexts (55). Notably, in patients with discordant imaging phenotypes, such as those with FDG-positive but PSMA-negative lesions, treatment response to ^177^Lu-PSMA is often suboptimal (56). Therefore, integrating ^18^F-FDG PET into baseline evaluation may allow clinicians to identify patients who would benefit from intensified or alternative therapeutic strategies, such as combination radiopharmaceutical and chemotherapy regimens.

Although imaging biomarkers derived from PSMA PET provide valuable information on tumor burden and tracer avidity, clinical and biochemical parameters remain of great value for a comprehensive understanding of patient prognosis. Parameters such as baseline PSA levels, circulating tumor DNA, alkaline phosphatase, lactate dehydrogenase, and hemoglobin concentrations have consistently demonstrated prognostic value in patients with mCRPC undergoing ^177^Lu-PSMA therapy (57). Integrating these clinical variables with imaging-derived metrics could result in robust multimodal prognostic models for ^177^Lu-PSMA therapy outcomes. Furthermore, combining imaging and clinical data in a unified analytic framework aligns with contemporary trends in radiomics and machine learning, where multiparametric datasets enable individualized outcome prognostication and real-time treatment adaptation.

Our findings suggest that potential quantitative PSMA PET/CT measurements, including TV, PSMA TL, and SUV_mean_, could serve as prognostic indicators for optimal patient selection in the future. However, several limitations should be acknowledged. First, the high heterogeneity observed in TV and TL analyses indicates variability in imaging protocols and patient selection across studies. Current data regarding standardized imaging protocols, optimal thresholds for SUVs, and the effect of the interval between the last therapy cycle and imaging remain insufficient and require further research. Concerning the assessed outcomes, additional adjustments may be necessary for disease stage and clinical symptoms, a factor not consistently accounted for across the included studies. Moreover, since most of the included studies were retrospective in design, their applicability for evaluating the effectiveness of these measurements for patient selection is limited, highlighting the need for validation through prospective studies. Also, relying solely on imaging factors for patient selection is inadequate. Multimodal approaches, including other factors, such as clinical and biochemical components, can eventually lead to developing robust guidelines for patient enrollment. Additionally, within anatomic site subgroups, different PET-derived biomarkers were pooled together, which may have introduced heterogeneity. Furthermore, publication bias was not formally assessed because of the insufficient number of studies per subgroup (<10), which may limit the completeness of the evidence synthesized. Future research is vital in exploring how integrating PET/CT findings into clinical decision-making can enhance patient outcomes.

## CONCLUSION

In this meta-analysis, we reviewed the prognostic value of PSMA PET/CT biomarkers in ^177^Lu-PSMA RPT for mCRPC. Although our results suggest SUV_mean_, TV, and TL can be promising candidates as predictors of survival and treatment response, further research is needed to optimize their real-life clinical application. Unified PET imaging techniques, timing, and value standardization, along with prospective validation and integration with clinical and laboratory findings, are essential for refining their prognostic utility and improving patient selection.

## DISCLOSURE

No potential conflict of interest relevant to this article was reported.

## Notes

KEY POINTS **QUESTION:** What is the prognostic value of quantitative PSMA PET biomarkers in assessing treatment response to ^177^Lu-PSMA therapy in patients with prostate cancer? **PERTINENT FINDINGS:** This systematic review and meta-analysis of 23 studies including 2,403 patients found that certain PSMA PET metrics, particularly TV and SUV_mean_, were associated with treatment outcomes. **IMPLICATIONS FOR PATIENT CARE:** Quantitative PSMA PET imaging biomarkers could help identify which patients are more likely to benefit from ^177^Lu-PSMA therapy, which might potentially improve personalized treatment planning in advanced prostate cancer.
